# Infrapatellar Fat Pad in Knee Osteoarthritis: A Comprehensive Review of Pathophysiology and Targeted Therapeutic Strategies

**DOI:** 10.3390/ijms262110408

**Published:** 2025-10-26

**Authors:** Ilenia Mallia, Antonella Fioravanti, Serena Guiducci

**Affiliations:** 1 Rheumatology Unit, Department of Clinical and Experimental Medicine, University of Florence, 50134 Florence, Italy; serena.guiducci@unifi.it; 2 Independent Researcher, 53100 Siena, Italy; fioravanti7@virgilio.it

**Keywords:** osteoarthritis, infrapatellar fat pad, adipose tissue, adipokines, pathophysiology, treatment

## Abstract

Osteoarthritis (OA) is the most common joint disorder globally, affecting approximately 595 million individuals and representing the first cause of chronic pain and disability. Recently, the infrapatellar fat pad (IFP), an intracapsular adipose tissue in the human knee joint, was recognized as an active and metabolically significant contributor to the pathophysiology of OA through the release of pro-inflammatory cytokines, adipokines, and growth factors that sustain inflammatory response, fibrotic remodeling, and neurogenic pain. The present review provides an overview of the pathophysiological significance of the IFP in OA and current and promising therapeutic strategies targeting this adipose structure. We summarize the available preclinical and translational evidence on conservative therapies, minimally invasive interventions, and surgical options as well as IFP-derived mesenchymal stromal cells as a potential cell source for cartilage repair. Overall, preclinical research indicates that the modulation of IFP inflammation and fibrosis could alleviate pain and delay the progression of the disease. The superficial location and its central role in the pathogenesis of OA make the IFP a promising therapeutic target in knee OA (KOA).

## 1. Introduction

Osteoarthritis (OA) is the most common joint disorder worldwide, and is recognized as a primary cause of chronic pain and disability in adults [1]. According to the data available from the Global Burden of Disease Study 2021, it has an impact on approximately 7.6% of the world’s population in 2020 (95% UI: 6.8–8.4%), affecting 595 million people (with a 95% uncertainty interval of 535–656 million) [2]. It is a degenerative joint disease involving the progressive disruption of articular cartilage, subchondral bone remodeling, synovial joint inflammation, and changes in periarticular structures [3].

Previously considered as a purely mechanical condition resulting from joint “wear and tear,” OA is currently recognized as a complex, multifactorial disease that affects all components of the joint, including articular cartilage, subchondral bone, ligaments, and surrounding muscles [4].

As defined by the Osteoarthritis Research Society International (OARSI), OA is characterized by joint impairment driven by cellular stress responses and the breakdown of the extracellular matrix [5]. The onset of the disease is determined by acute injuries or repetitive microtraumas which break the homeostatic balance of joint tissues leading to metabolic dysregulation, and may interact with genetic susceptibility and other environmental factors [6]. A modern approach to understanding OA reconsiders the Infrapatellar Fat Pad (IFP) and the adjacent synovial membrane (SM) as a single anatomo-functional unit (AFU) [7]. Several lines of evidence, ranging from macroscopic anatomy, imaging, histopathology, and molecular biology highlight the strict interaction between these structures [8,9,10]. The anatomical proximity and molecular interactions suggest a “mutual conditioning,” where pathological changes in one tissue influence the others [11]. This AFU is actively involved also in the pathogenesis and pain of OA, with histopathological analyses confirming that both the IFP and SM show increased inflammatory cells and increased vascular density in OA [7].

Studies show that low-grade chronic inflammation emerges as a key driver in the pathophysiology of OA, affecting not only the cartilage but also the joint structures and surrounding tissues [12]. Elevated levels of pro-inflammatory cytokines such as Interleukin (IL)-1β, Tumor Necrosis Factor (TNF)-α, and IL-6 in joint tissues promote the expression of catabolic enzymes, including matrix metalloproteinases (MMP) and aggrecanases, as well as molecules like nitric oxide (NO) and Prostaglandin E2 (PGE2), which collectively degrade the ECM and inhibit cartilage repair [13,14]. Synovitis is a frequent finding in OA and results from the activation of synovial cells by damage-associated molecular patterns (DAMPs), stimulating cytokine production and immune cell infiltration [15]. Oxidative stress exacerbates these inflammatory processes enhancing pro-inflammatory signaling and inducing chondrocyte apoptosis and senescence [16]. Furthermore, emerging evidence suggests that alterations in gut microbiota composition may disrupt host–microbe homeostasis, provoking immune activation and stimulating the gut–joint axis, thereby playing a role in the development of OA [17].

Taken together these events promote progressive morphological alterations such as degradation of cartilage, remodeling of subchondral bone, formation of osteophytes, and inflammation of the SM, ultimately leading to the loss of normal joint function and structural integrity.

Although any synovial (diarthrodial) joint could be affected, hips, knees, and interphalangeal and metacarpophalangeal joints of hands are most commonly affected sites [18,19]. The pathogenesis of the disease is multifactorial, influenced by systemic and local factors. Aging is the strongest non-modifiable risk factor, associated with cellular senescence and impaired cartilage matrix repair [3,20]. Genetic predisposition also plays a crucial role, suggesting a substantial genetic component to OA susceptibility, and the disease shows a higher prevalence in women, particularly after menopause [21]. Other significant factors include a history of major joint trauma (post-traumatic OA), overuse, and pre-existing anatomical abnormalities such as joint malalignment or dysplasia [22].

Among the modifiable and local risk factors, obesity is a major contributing factor through complex biomechanical and metabolic mechanisms acting at both systemic and local tissue levels [23,24]. Fatty tissue functions as a metabolically active secretory organ rather than an inert lipid reservoir, promoting the secretion of pro-inflammatory mediators, regulatory hormones, and additional bioactive mediators, collectively referred to as adipokines [25,26]. These secreted substances modulate critical biological functions including metabolic homeostasis, immune responses and they are critically involved in the regulation of bone remodeling, exerting their effects through endocrine, paracrine, and autocrine pathways [27,28]. They mediate the activity of osteoblasts and osteoclasts, influencing the dynamic equilibrium between bone formation and resorption and affecting cartilage metabolism [29]. Leptin, for instance, has been shown to exert both anabolic and catabolic effects on bone, depending on whether it acts centrally via the hypothalamus or peripherally on bone cells [30]. It induces chondrocytes to produce pro-inflammatory cytokines, adhesion molecules and matrix-degrading enzymes, thereby accelerating ECM breakdown [31]. Adiponectin shows a pro-osteogenic effect during bone remodeling [32,33] and inhibits osteoclastogenesis [34]. Although sometimes linked to bone-protective effects, it also induces the release of interleukins, MMPs and nitric oxide (NO) in chondrocytes, which can promote cartilage damage [35,36]. Other adipokines, such as resistin and visfatin, have been implicated in promoting a pro-inflammatory environment that may enhance osteoclast activity and bone resorption [37,38]. Visfatin, highly expressed in the IFP near osteophyte formation, reinforces local inflammation and enhances chondrocyte production of cytokines and chemokines, contributing further to cartilage catabolism [39,40].

IFP, synovium and osteophyte cells are active producers of inflammatory cytokines and adipokines [10]. These molecules, in particular leptin and adiponectin, can upregulate inflammatory mediators including PGE2, IL-6 and IL-8, TNF-α, and vascular cell adhesion molecule 1 (VCAM-1) within the synovial fluid of the knee [41,42] modulating the infiltration of inflammatory cells into cartilage, finally inducing a degenerative cascade that exacerbates the progression of OA. Correspondingly, studies have demonstrated that adipokine concentrations are markedly elevated in individuals with OA, and these increased levels remain significantly correlated with disease severity even after adjusting for confounding factors such as age, sex, and Body Mass Index (BMI) [43,44].

The dysregulation of adipokine signaling promotes chronic low-grade inflammation, which collectively accelerate cartilage degradation through upregulation of MMP and aggrecanases [45]. Furthermore, obesity-associated metabolic syndrome factors, including insulin resistance and dyslipidemia, contribute to synovial inflammation and chondrocyte dysfunction through advanced glycation end-product formation and oxidative stress pathways [46,47].

While this review primarily focuses on human KOA and findings from murine preclinical models, we need to consider the significant contribution of the canine model to our understanding of OA. A key study of Schmidli et al. investigated the inflammatory activity of the IFP in dogs with cranial cruciate ligament rupture (CCLD), a condition that leads to OA [48]. The results demonstrated that the IFP of dogs with CCLD presented a significant increase in immune cells, particularly T lymphocytes (CD3) and macrophages (CD14), compared to healthy dogs [48]. Additionally, increased gene and inflammatory markers levels, such as IL-1β, IL-6, MMP-1, and MMP-13, were found [48]. Adipokines analysis revealed increased adiponectin and decreased leptin secretion in the IFP of dogs with CCLD compared to controls [48]. As one of the earliest and most consistently observed abnormalities following a CCLD tear in dogs, the fat pad sign, characterized by the presence of edema on MRI (visualized as hyperintensity on T2-weighted sequences), volumetric increase, and fibrosis is able can be detected in inflamed or irritated Hoffa’s fat pad [49]. These data indicate that IFP is a potential contributing factor to the pathogenesis of chronic joint disease, due to its inflammatory phenotype and its proximity within the knee joint.

This review provides a comprehensive analysis of emerging therapeutic options targeting the IFP in Knee Osteoarthritis (KOA), examining their effects on inflammatory factors, fibrotic pathways and nervous sensitization. We combine preclinical and translational data to provide evidence of the central role of the IFP in OA pathophysiology, discussing future directions for clinical application.

### Pathophysiological Roles of the Infrapatellar Fat Pad in OA

**Figure 1:**
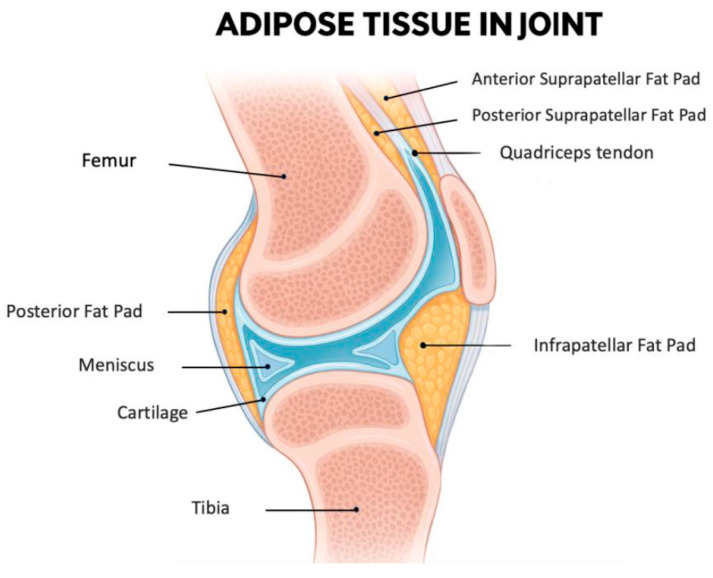
A midsagittal section of the knee joint illustrates the anatomical position of the IFP, along with the other smaller adipose structures: the posterior fat pad, the anterior suprapatellar fat pad, and the posterior suprapatellar fat pad. The IFP is located inferior to the patella and posterior to the patellar tendon. Anteriorly, it is enclosed by the joint capsule, while its articular-facing surface is lined by synovial membrane. As such, the IFP is included within the joint capsule (intracapsular) but outside the synovial cavity (extrasynovial). Additionally, it lies in close proximity to the articular cartilage surfaces, highlighting its potential functional significance in joint biomechanics. Figure created with the assistance of artificial intelligence and scientifically validated by the authors.

The IFP, also known as Hoffa’s fat pad, is an intra-articular but extra-synovial adipose tissue situated in the anterior region of the knee joint (Figure 1). This specialized fibrofatty tissue is strategically located between the patellar ligament and SM extending from the inferior patellar pole to the tibial tuberosity [50]. Functionally, it is a complex biomechanical structure that serves as a sophisticated cushion that optimizes joint movement by dynamically accommodating synovial fluid redistribution throughout the flexion-extension cycle [51]. Being an intra-articular fat depot, it is in direct anatomical continuity with synovial and cartilaginous structures making it particularly relevant to joint homeostasis.

**Figure 2:**
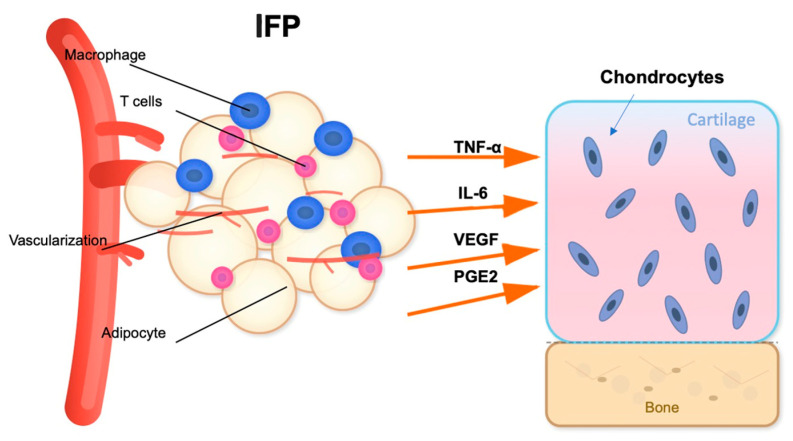
Inflammatory signaling pathway between the IFP and articular cartilage. The IFP contains adipocytes, macrophages, and T cells within a highly vascularized environment. Upon activation, these cellular components release pro-inflammatory cytokines including TNF-α, IL-6, VEGF, and PGE2. These inflammatory mediators target the articular cartilage, potentially affecting chondrocyte metabolism and contributing to cartilage degradation [53]. The proximity of the IFP to the cartilage-subchondral bone interface facilitates this paracrine signaling mechanism in knee joint pathophysiology. Figure created with the assistance of artificial intelligence and scientifically validated by the authors.

In addition to its mechanical function, the IFP acts as a metabolically active organ and a major player in obesity-related OA pathogenesis [52]. The IFP is a significant source of cytokines (IL-6, IL-1β, TNF-α), adipokines (leptin, resistin, adiponectin), and growth factors (like vascular endothelial growth factor, VEGF) [53] (Figure 2). Aging and obesity promote adipocyte hypertrophy and immune cell infiltration, especially pro-inflammatory M1 polarized macrophages and T cells, which contribute to local inflammation [54]. Simultaneously, the collection of senescent cells in the IFP drives the senescence-associated secretory phenotype (SASP) [55], releasing matrix-degrading enzymes and enhancing cytokine networks. A comparative study of IFP tissue from healthy young and elderly individuals found that aging is associated with distinct changes in adipocyte morphology and the ECM [56]. Specifically, adipocytes in the elderly group showed a significant increase in area and volume than younger one indicating age-related hypertrophy. Concurrently, the ECM in the older group was characterized by lower total collagen and a reduction in elastic fibers [56]. These physiological aging processes alter the cellular and structural composition of the IFP, which may compromise its biomechanical properties and contribute to the low-grade inflammatory state known as “inflammaging.” However, the structural changes in IFP, particularly regarding volume, remain a subject of debate with conflicting findings. Some studies suggest an increase in IFP volume in OA patients, potentially driven by local inflammation, adipocyte hypertrophy, and neovascularization, often correlating with higher BMI [57,58]. In contrast, other research reports a decrease in IFP volume or no significant change in OA, a phenomenon that could be attributed to the progressive development of fibrosis, leading to tissue contraction and atrophy in more advanced stages of the disease [59]. This discrepancy highlights the complex nature of IFP, suggesting that its volume may change dynamically depending on the stage of OA and the underlying pathological processes.

Biological and biomechanical changes in IFP may also negatively impact adjacent synovium and cartilage via paracrine effects, eliciting synovitis and promoting cartilage degradation [60,61].

IFP can contribute to fibrotic remodeling, one of the main changes in OA, enhancing collagen deposition [10,62].

Remodeling is the final result of interconnected mechanisms, with Transforming growth factor-β (TGF-β) signaling pathways being the primary secretary, and pro-fibrotic M2 macrophages and constant low-grade inflammation offering a continuous stimulus for matrix production [63]. The fibrotic remodeling changes the essential biomechanical features of the IFP, alters its compliance, compromises its capacity to absorb joint stress effectively, and accelerates the deteriorative changes in the whole compartment [64]. Experimental results verify this mechanism demonstrating that post-inflammatory irreversible structural changes in the IFP plays a key role in sustaining chronic pain in rat OA models [65].

The neurogenic inflammation within the IFP also plays a central role in mediating OA pain [66]. The IFP is densely innervated by sensory and autonomic nerve fibers, especially by branches of the tibial and peroneal nerves [50]. In OA, the IFP shows increased expression of substance P and calcitonin gene-related peptide (CGRP), which play an important role in pain transmission and neurogenic inflammation [67,68]. Histological analysis showed an increase in the number of CGRP-positive nerve fiber endings in fibrous IFP, especially in the areas of neovascularization [69], with further increase as the OA progresses [70]. Additionally, IFP contains substance-P sensory nerve fibers that promote macrophage activation, which subsequently release of pro-inflammatory cytokines, such as IL-6 [66].

Recent research highlighted the role of mechanosensors, particularly the Piezo1 and Piezo2 ion channels, in mediating both mechanoceptive and inflammatory stimuli able to influence knee pain in OA [71]. Indeed, Piezo channels have been found to be involved in the inflammatory pathways of KOA, mediating the inflammatory response in chondrocytes and synovial cells [72]. Excessive mechanical stress, a risk factor for OA, upregulates Piezo1 expression, leading to a calcium influx that activates inflammatory responses and can further sensitize the channels, creating a pathogenic feedback loop [7]. Piezo2, in particular, is the most implicated in mechanical sensitization and pain responses, with higher immunoreactive vessel density in the IFP and SM compartments of the AFUs in OA compared to healthy subjects [7].

The presence of synovitis is associated with diminished pressure pain thresholds (PPTs) of the patella on Magnetic Resonance Imaging (MRI), which indicates an increased pain sensitivity and diminished pain functionality [73]. Synovitis has been shown to be significantly associated with reduced PPTs at the patella, showing an increase in local pain sensitivity [73]. This finding suggests that synovial inflammation contributes to peripheral sensitization, in which nociceptive neurons in the joint become hyper-responsive to mechanical stimuli. The lowering of PPTs indicates a lower tolerance to pressure-induced pain, which hesitates into impaired pain inhibitory mechanisms and reduced functional capacity in patients with joint pathology [74]. These changes in nociceptive processing are consistent with findings from previous studies linking synovial inflammation to clinical pain severity and structural progression in OA [75,76]. Furthermore, the role of synovitis in amplifying nociceptive signaling aligns with evidence showing that inflammatory mediators released within the SM, such as cytokines and prostaglandins, can directly sensitize joint afferents, thereby enhancing the subjective experience of pain [77,78]. The coexistence of inflammation, fibrosis and neo-innervation create the condition for a maladaptive pain amplification circuit potentially leading to a critical transition from acute-to-chronic OA pain while increasing overall nociceptive burden typical of knee KOA [79].

All the above-mentioned mechanisms are combined with its subcutaneous localization, making IFP an easily accessible site for minimal invasive interventions, a newer therapeutic target to treat KOA.

## 2. Therapeutic Strategies Targeting the Infrapatellar Fat Pad in Osteoarthritis

In the following sections, we will delineate the currently available therapeutic options targeting the IFP in KOA, ranging from conservative (Table 1) to minimally invasive (Table 2) and surgical treatments (Table 3). 

### 2.1. Conservative Therapies

#### 2.1.1. Anti-Inflammatory Agents

When comparing the inflammatory profile of the IFP from patients with KOA to subjects with traumatic knee injuries, such as an anterior cruciate ligament (ACL) rupture, distinct patterns related to inflammation are exhibited [107]. Studies showed that the IFP from OA patients is significantly more inflamed and vascularized than the IFP from patients with ACL injuries [107].

Furthermore, a study of Timur et al. demonstrated that the IFP from OA patients secretes significantly higher levels of prostanoids (PGE2, PGF2α, and PGD2) compared to IFP from subjects with cartilage defects [80], suggesting a distinctive inflammatory profile associated with OA pathogenesis. The secretion pattern of prostanoids in OA IFP samples can be stratified into two distinct subgroups: one characterized by markedly elevated prostanoid levels indicative of heightened inflammatory activity, and the other showing comparatively moderate reductions in prostanoids production [80]. This heterogeneity in prostanoids secretion reflects varying degrees of inflammatory microenviroment within the joint and may influence disease severity and symptoms [80]. Notably, the selective COX-2 inhibitor celecoxib has been shown to exert anti-inflammatory effects in both subgroups, primarily through the suppression of prostanoids synthesis by the IFP [80]. The efficacy of celecoxib appears particularly pronounced in individuals with higher baseline prostanoids secretion, underscoring its role in modulating inflammation mediated by lipid-derived mediators in OA [80]. These findings not only highlight the IFP as a dynamic source of pro-inflammatory prostanoids but also support targeted therapeutic strategies aiming to modulate local inflammatory pathways to alleviate joint inflammation and pain.

#### 2.1.2. Low-Intensity Pulsed Ultrasound on the Fibrosis of the IFP

Low-Intensity Pulsed Ultrasound therapy (LIPUS) has demonstrated efficacy in alleviating KOA discomfort [108].

According to thermogenic mechanisms and high-frequency oscillatory stimulation, LIPUS exposure results in reduced edema and nociception and increased function [81,109].

Research conducted by Kitagawa and colleagues [110] demonstrated that LIPUS attenuated IFP fibrosis via multiple pathways. It inhibits Hypoxia Inducible Factor-1 (HIF-1) function, which correlates with macrophage phenotypic transformation [111], promoting M2 macrophage differentiation while reducing M1 phenotype (pro-inflammatory) prevalence. Furthermore, LIPUS has been documented to increase macrophage populations [112], decreases macrophage migration into the SM and reduce pro-inflammatory mediator release [113], showing a regulatory mechanism that promote modulation of macrophage activity through these combined effects [114].

Additionally, LIPUS leads to the downregulation of the genetic transcription of TGF-β [115], suppressing TGF-β-mediated fibrotic processes in the cultured synovial fibroblasts [116], inhibiting the osteoclastogenesis through the interference of the TGF-β1/Smad3 pathway [117], and subsequently the subchondral bone resorption.

#### 2.1.3. Gene Therapy

As previously mentioned, fibrosclerosis of Hoffa’s fat pad has a central role in the pathogenesis of degenerative knee arthropathy through histiocytic invasion, angiogenesis, and expansion of nociceptive innervation, resulting in persistent pain [69].

Monocyte chemoattractant protein-1 (CCL2/MCP-1), a strong chemotactic factor for histiocytes, shows increased expression in various fibrotic disorders like hepatic cirrhosis [118], nephrosclerosis [119], and progressive systemic sclerosis [120].

The CCL2/C-C Motif Chemokine Receptor 2 (CCR2) signaling pathway is the most frequently documented mechanism responsible for the recruitment of circulating monocytes in OA [121,122] as well as for pain development in murine OA models [123]. Elevated levels of CCL2 have been observed in the IFP, synovial tissue [10,62] and blood serum of individuals with OA [124]. N-terminally truncated or chemically altered CCL2 variants have been identified as functional receptor antagonists [82]. Research suggests that targeting CCL2 could be a promising strategy to inhibit osteoclast formation, as the 7ND variant has been shown to prevent human osteoclast differentiation [125]. In addition, administration of 7ND markedly decreased osteolysis induced by wear particles and reduced both inflammatory cell infiltration and osteoclast numbers [126].

In a study conducted by Yoshimura et al. [82], employing a rat inflammatory arthropathy model, gene delivery of 7ND significantly suppressed the histological fibrosis in the IFP, decreased macrophage infiltration in synovial tissues, and led to a notable reduction in local CCL2 levels, apparently produced by infiltrating macrophages, ultimately inhibiting CCL2-mediated macrophage migration, and lowering the production of inflammatory cells and additional chemokines in the IFP.

Targeting CCL2 appears to play a central role in mitigating IFP fibrosis and limiting the inflammatory activation process [82]. Nevertheless, the precise in vivo mechanisms through which 7ND exerts its antagonistic effects still remain insufficiently understood [127]. Additional investigations are required to fully clarify biological pathways influenced by 7ND.

#### 2.1.4. Diet

Several studies, using high fat-diet-induced murine OA models, reported that IFP volume or area was increased after administration of lipid-enriched diet [128,129,130].

In humans, however, the association between systemic anthropometric indices, such as BMI, and the size of the IFP is more complex, with conflicting results across studies. Some research, for instance, did not find a significant correlation between measures of adiposity and IFP volume in either OA patients or control subjects [131,132], suggesting that local joint factors may have a greater influence than systemic body composition in determining IFP size. In contrast, other studies have identified a positive relationship [133], although this can be influenced by factors such as sex [134]. For example, a significant correlation between IFP volume and body mass has been observed in females but not in males, highlighting potential sex-specific differences in adipose tissue metabolism within the knee [134]. The conflicting aspects of these findings may be attributed to differences in study methodologies, patient populations (including OA severity and demographics), and imaging techniques.

Radakovich et al. [83] indicated that the size of IFP was not dependent on body weight in the guinea pigs exposed to different dietary conditions. At the same time, the gene expression profiles within the IFP significantly varied. Specifically, this study showed higher transcription of pro-inflammatory genes (like Nuclear factor kappa light chain enhancer of activated B cells, NF-κB) in the IFP of hyperlipidic diet-fed guinea pigs than in the obese guinea and hypocaloric standard diet-fed pigs, enhancing expression of inflammatory signaling molecules including IL-1, IL-6, and TNF-a that may directly act on pathologic processes related with inflammation [83,84]. In that context, the COX-2 cascade and MCP-1 which is known to stimulate MMP activity, were overexpressed in hyperlipidic diet cohort inducing breakdown of type II and IV collagen within articular cartilage [83].

Taken together, this evidence demonstrated that immune stimulation is greater in the knee joint of obese and hyperlipidic diet groups versus lean. In addition, these data support the concept that low-caloric diets with a low-fat content result in reduced knee joint inflammation.

#### 2.1.5. Exercise

It has been established that the IFP exhibits dynamic movement during knee joint motion [135]. Several studies suggest that the IFP plays a functional role promoting efficient joint movement, maintaining joint space, and acting as a cushion to absorb mechanical loads, thereby protecting the joint structures [135,136].

Histological analyses in knee immobilization in rat models [137,138] and in OA rat models induced by monoiodoacetate [85], demonstrated pathological changes in the IFP, including adipose tissue atrophy, fibrosis, and vascular congestion, particularly in the anterior region [85].

Although there is no direct evidence on the combined effect of joint mobilization and range-of-motion (ROM) exercises on adipose tissue atrophy and the structural integrity of the IFP, studies suggest that such interventions may improve joint function and positively influence the surrounding tissues.

In particular, a study of Griffin et al. showed, using murine models, that while starting of daily exercise temporarily triggered inflammation in the synovium and IFP along with changes in tissue architecture, continued daily exercise supported the recovery and maintenance of IFP homeostasis. Takeda et al. [86], also using murine models, demonstrated that joint movement not only reduced adipose cell degeneration in the IFP compared to non-treated controls, but may also exert a prophylactic effect. Nonetheless, the precise mechanisms underlying these therapeutic effects remain unclear and warrant further investigation to optimize treatment strategies.

### 2.2. Minimally Invasive Treatments

#### 2.2.1. Intra-Articular Injective Therapies

Hyaluronic acid (HA), a macromolecular glycosaminoglycan, is a key substance produced by the SM and present in hyaline cartilage [139]. Intra-articular administration of HA provides mechanical viscosupplementation to articular surfaces, diminishes cartilaginous degradation, promotes trophic support to chondrocytes, and enhances synthesis of native hyaluronan, consequently slowing the progression of OA [87,88].

Studies of Chen and Qu [140,141] collectively highlight that targeting the IFP with HA can effectively modulate local inflammation and promote both structural preservation and symptomatic relief in OA. In particular in the study of Chen et al. [141] the researchers found, employing an in vitro model, that treatment with a combination of HA and platelet-rich plasma (PRP) significantly reduced the secretion of pro-inflammatory cytokines and adipokines from the IFP adipocytes. This anti-inflammatory effect helped to restore chondrocytes’ ability to produce a cartilage-like ECM, suggesting that HA can positively influence the local joint environment and protect cartilage by modifying the behavior of inflamed IFP cells [141]. Study of Qu [140] extended this concept into an in vivo setting using a rat model of OA. Here, HA was applied in the form of a biodegradable sheet placed directly onto the IFP. The treatment led to a marked reduction in fibrotic tissue remodeling and nerve fiber ingrowth within the fat pad, both known contributors to pain in OA. Together, these studies suggest that HA does more than lubricate the joint; whether delivered as an injectable compound (with PRP) or as a physical implant (in sheet form), HA appears capable of altering the IFP’s pathological signaling in OA, offering a potential disease-modifying and analgesic effect.

Platelet-rich plasma (PRP): Recent advances in tissue biology elucidated the crucial role of growth factors (GFs) in maintaining tissue homeostasis and orchestrating reparative responses to pathological insults, with extensive in vitro and in vivo studies examining their impact on chondral regeneration [142]. PRP intra-articular injection causes an initial burst, then a sustained release of biologically active substances, including key growth factors such as platelet-derived growth factor (PDGF), TGF-β, insulin-like growth factor I (IGF-I), and VEGF [89]. These signaling proteins play essential roles in tissue repair processes, including the prevention of chondrocyte apoptosis, promotion of angiogenesis and osteogenesis, regulation of the inflammatory response, and stimulation of collagen production [143,144]. Moreover, additional components released by platelets, such as fibrin, contribute to tissue regeneration by serving as both a structural matrix and a chemoattractant, facilitating the recruitment of stem cells and other reparative cell populations to the site of injury [145,146].

Research conducted by Araya and colleagues [90] revealed that intra-articular administration of pure PRP can reduce pain and suppress the advancement of synovial inflammation, infrapatellar adipose tissue architectural alterations, and articular cartilage deterioration in the short-term period. Furthermore, it was detected a reduction in the expression of CGRP nerve fibers in IFP of KOA patients treated with pure PRP [90]. The diminished presence of CGRP-positive nerve fibers after PRP treatment suggests that PRP has the ability to modulate the local neural environment mitigating nociceptive sensitization. Notably, this effect appears to be independent of the degree of cartilage damage within the knee joint [147]. This observation is consistent with findings from prior studies, which have similarly indicated that PRP can exert analgesic effects through mechanisms beyond cartilage repair [148,149].

Corticosteroids (CCS): Steroids modulate immune cells, as they regulate the polarization of macrophages, promoting immune cells switching from the pro-inflammatory M1 phenotype into an anti-inflammatory M2 phenotype [91] and inhibiting T-cell activity and proliferation [92]. Moreover, CCS also have powerful anti-catabolic effects as they inhibit the production of MMPs and other proteolytic enzymes responsible for cartilage degradation [93].

The analgesic efficacy of CCS in IFP-related pathology acts through multiple mechanisms, including the suppression of inflammatory mediator production, attenuation of peripheral nerve sensitization, and modulation of nociceptive signal transduction [150,151]. Additionally, these therapeutic agents exhibit anti-fibrotic properties by interfering with TGF-β signaling pathways, suggesting their potential role in reducing excessive collagen deposition and promoting favorable tissue remodeling within the fat pad [152,153]. This multifaceted mechanism of action positions CCS as valuable therapeutic interventions for addressing both the inflammatory and structural alterations characteristic of IFP pathology in OA conditions.

Research conducted by Heard and colleagues [103] revealed that in a surgically induced IFP impingement, a single intra-articular injection of CCS administered during the operative procedure effectively attenuated the acute inflammatory response within the articular space, primarily by suppressing cellular proliferation. However, this intervention proved inadequate for sustaining joint protection through the 9-week postoperative period.

It was also demonstrated that MPA (Methylprednisolone acetate), in ovine model, attenuated the IL1β-mediated transcriptional upregulation of MMP genes within the hyaline cartilage of various joint surfaces (including the kneecap and trochlear groove, distal femoral articular surfaces, and proximal tibial articular surface), synovial tissue, and IFP, demonstrating a dose-dependent suppressive effect [104].

The GLITTERS randomized controlled trial, the first trial that investigated corticosteroid administration directly into the IFP rather than the intra-articular space, failed to demonstrate favorable outcomes [154]. The study revealed that targeted corticosteroid infiltration of the infrapatellar adipose tissue showed no significant efficacy in mitigating articular pain or diminishing the volume of effusion-synovitis in patients with inflammatory KOA [154]. Nevertheless, the emergence of encouraging trends warrants further investigation through large-scale, multicentric clinical trials to comprehensively evaluate the therapeutic potential and clinical relevance of this intervention.

C-type natriuretic peptides (CNP): C-type natriuretic peptide belongs to the natriuretic peptide superfamily and has been documented to suppress the transcriptional activity of type I collagen in both pulmonary alveolar epithelial cells and cardiac myocytes through downregulation of the TGF-β signaling cascade [155,156].

Research conducted by An and colleagues [94] demonstrated that the administration of CNP via intra-articular injection effectively inhibited fibroproliferative alterations within the IFP, resulting in significant mitigation of chronic knee pain. Furthermore, local articular administration of CNP was shown to attenuate the progressive deterioration of hyaline cartilage [94]. These findings indicate that CNP represents a promising disease-modifying OA drug.

#### 2.2.2. Electroacupuncture

Electroacupuncture (EA), extensively used in clinical settings, involves the application of pulsed electrical stimulation to acupuncture points following needle insertion [157]. This non-invasive therapeutic modality is broadly employed in the management of KOA, with various acupuncture techniques having demonstrated clinical efficacy [158,159]. As a conventional intervention for pain management, its therapeutic properties including analgesia, sedative effects, enhancement of circulatory function, and modulation of muscular tone have been validated through extensive clinical evidence, leading to improvement of joint pain, edema and functional mobility associated with KOA [95,96,97].

Research conducted by Zhang and colleagues [98] revealed that EA not only mitigated cartilaginous deterioration but also attenuated synovial inflammatory processes and IFP fibrotic changes, suppressing NLR family pyrin domain containing 3 (NLRP3) inflammasome activation, thereby reducing the inflammatory microenvironment characteristic of KOA.

#### 2.2.3. Genicular Artery Embolization (GAE)

The primary blood supply to the IFP originates from branches of the genicular arterial system, which penetrate the tissue and form an extensive network of anastomotic connections throughout the structure [160]. This rich periarticular vascular network surrounding the knee joint is clinically significant, as it influences the fat pad’s response to injury, its healing potential, and its involvement in various pathological conditions affecting the knee [161]. Within this perspective, GAE has been introduced during the last ten years as an innovative percutaneous intervention for managing pain associated with degenerative knee joint disease [99]. This procedure specifically targets the abnormal neovascularization in arthritic knee joint, a vascular network believed to contribute to inflammatory processes, promoting nociception and synovial hyperplasia [162]. Through the selective obstruction of pathological branches of the genicular vessels, GAE diminishes blood flow to the synovium and reduce inflammatory activity, consequently providing pain relief while preserving the mechanical integrity of the joint [99,100].

A study of Sun et al. [163] evaluated the safety and efficacy of GAE in 33 patients with mild to severe KOA. At 12-month follow-up, the procedure demonstrated a significant reduction in pain and improvement in function in both patients with mild-to-moderate and severe OA, showing that GAE is a well-tolerated and effective treatment for improving symptoms and function in this kind of patients.

GAE shows also a favorable safety profile over a two-year follow-up period [164]. The most common adverse events are minor and self-limiting, primarily consisting of temporary skin discoloration and one groin hematoma, making it a promising therapeutic option for patients where surgery is contraindicated [164].

However, therapeutic efficacy is not uniformly achieved [163]. Therefore, careful candidate identification and appropriate prognostic counseling remains crucial.

#### 2.2.4. Genicular Nerve-Targeted Cooled and Pulsed Radiofrequency Ablation

The IFP is richly innervated, creating a complex neural network that contributes significantly to knee pain experience in KOA patients. The fat pad is primarily innervated by branches of the femoral, common peroneal, and saphenous nerves, with particular contribution from the infrapatellar branch of the saphenous nerve and articular branches that form part of the genicular nerve complex. [50,165]

The dense neural supply of the IFP includes both sensory and sympathetic nerve fibers, containing numerous nociceptors and mechanoreceptors that make it particularly sensitive to mechanical stress and inflammatory mediators [68]. In KOA, the fat pad often becomes fibrotic, inflamed, and hyper innervated, leading to enhanced pain transmission and contributing to the overall pain experience [69]. Radiofrequency ablation has emerged as promising interventional approach for managing chronic knee pain by targeting the neural structures innervating the joint, including those supplying the IFP [166,167].

The technique involves using thermal or pulsed energy to disrupt nerve conduction, thereby interrupting pain signal transmission [101,102].

Conventional radiofrequency ablation creates thermal lesions at temperatures exceeding 80 °C [168], while cooled radiofrequency maintains tissue temperatures around 60–70 °C through internal probe cooling, allowing for larger lesion volumes [169].

A systematic review and meta-analysis by Soetjahjo et al. found that both cooled and pulsed Radiofrequency Ablation (RFA) techniques targeting genicular nerves provided significant pain reduction in KOA patients at all follow-up intervals (1, 3, 6, and 12 months post-treatment), with no significant difference in analgesic effectiveness between the two methods [170]. Pain scores, measured using Visual Analog Scale (VAS) or Numeric Rating Scale (NRS), showed substantial improvement particularly at the 6-month mark [170]. The meta-analysis revealed also that both techniques were generally safe with minimal adverse events reported, including only minor complications such as injection site pain, numbness, and stiffness that resolved quickly [170]. Both techniques offer effective pain relief for KOA patients for at least 6 months, though the long-term benefits beyond 12 months remain uncertain, possibly due to nerve regeneration [170].

This suggests that these non-conventional RFA techniques provide a valuable minimally invasive treatment option for managing chronic KOA pain when conservative therapies have failed.

### 2.3. Surgery

Evidence demonstrates that specific anatomical regions within the IFP, specifically the upper and lower sections, exhibit heightened vulnerability to biomechanical stress and loading forces during articular motion [171]. Such focal loading patterns induce hypoxic tissue damage, inflammatory responses, and promote releasing of inflammatory mediators and adipose-derived factors, thereby intensifying the manifestations of KOA [172].

The significant nociceptive innervation throughout the IFP also makes these compression zones primary generators of articular pain, perpetuating the pathological cascade [171].

Studies demonstrated that selective removal of the IFP, whether through partial or total excision, successfully diminished articular inflammation while preserving chondral structure, thereby slowing the progression of KOA [105,106].

Notably, targeted excision of adipose tissue enhanced biomechanical stability of the joint, maintained the structural integrity of the subchondral osseous tissue, and diminished degradation of the articular surface. Furthermore, immunohistochemistry evaluation revealed significant suppression of pro-inflammatory mediators, including IL-6, TNF-a, and MMP-3, alongside optimized retention of type II collagen [173].

The best approach to the IFP during total knee arthroplasty in patients with KOA remains controversial. A systematic review by Yao et al. [174] found inconsistent evidence regarding whether IFP resection leads to significantly inferior outcomes compared to preservation. Subsequently, Rajbhandari et al. [175] reported that IFP resection yielded marginally superior patient-reported functional outcomes as measured by Oxford Knee Scores; however, no significant difference in patient satisfaction was observed between the resection and preservation groups based on SF-12 scores.

## 3. The Use of Infrapatellar Mesenchymal Stromal Cells in Joint Cartilage Repair

Multipotent stromal cells (MSCs) exhibit immunomodulatory and nutritive properties involving anti-inflammatory, vasculogenic, and fibrosis-inhibiting mechanisms [176].

Research demonstrated that progenitor cells exhibiting stem cell properties are found within IFP, sharing characteristics with, though not being identical to, mesenchymal stromal cells of bone marrow origin [177].

These Infrapatellar Fat Pad-derived Stem Cells (IFPSCs) demonstrate reparative capacity and immunomodulatory properties, modulating macrophage phenotypic polarization, while exhibiting superior cartilage-forming potential compared to other mesenchymal stromal cells [177,178,179] due to their proximity to the knee joint and similarity to subcutaneous adipose tissue cells.

However, the therapeutic use of autologous stem cells shows significant challenges as the pathological state of the donor tissue is a critical factor. There is compelling evidence that IFP-SCs isolated from patients with advanced OA can exhibit a primed pro-inflammatory phenotype [180]. These cells may present significant expression of HLA-DR anti Fas/FasL, and a null expression of the CD38/NADase gene, indicating that the stem cells are from IFP of OA subjects in a chronic state of immune activation, without a substantial counteracting [180]. This characteristic could potentially undermine their regenerative function or even exacerbate the inflammatory environment within the OA joint if used without pre-treatment or appropriate cell selection. Despite this challenge, the promising therapeutic properties of IFP-SCs have driven their assessment within clinical contexts. Several early-phase clinical trials have been conducted to establish the safety and efficacy of implanting autologous IFP-derived MSCs for treating KOA and chondral defects [179,181]. These initial studies have generally reported positive outcomes regarding safety, pain reduction, and functional improvement, laying the groundwork for further research [182].

The mesenchymal-derived extracellular vesicles from the infrapatellar adipose tissue (MSCIPFP-Exos) mitigated OA progression in vivo through suppression of apoptosis, induction of ECM production, and downregulation of degradative mediators in vitro [183]. Furthermore, MSCIPFP-Exos substantially elevated autophagic activity within cartilage cells, mediated in part through microRNA 100-5p (miR100-5p)-dependent suppression of mTOR autophagy signaling cascade [183].

For these reasons, the stromal cells of IFP tissue have been focused on as potential therapeutic cell source for localized chondral defect in knee joint due to their anatomical location and surgical accessibility [184,185]. Nonetheless, although promising, its adoption into mainstream clinical practice is still in the preliminary stages. Key questions regarding optimal cell dosage, long-term efficacy, and strategies to mitigate the pro-inflammatory potential of cells from OA donors must be addressed through larger, well-controlled randomized clinical trials.

While MSCs often show poor adhesion, migration, and survival post-injection, a study of Yang et al. [186] demonstrates that using tropoelastin (TE) as an injection medium significantly improves IPFP-MSC adhesion, migration, and chondrogenic differentiation compared to standard carriers (normal saline, hyaluronic acid, platelet-rich plasma). TE also boosts ECM production in co-cultured osteoarthritic chondrocytes [186].

Moreover, when cultured within a HA-enriched environment, IFPSCs exhibit significantly enhanced chondrogenic differentiation capacity, characterized by increased expression of cartilage-specific genes and improved ECM synthesis [187].

Consequently, combined use of an HA-enriched microenvironment and IFPSC-based therapeutic strategies could improve the quality and long-term efficacy of joint cartilage repair, especially in degenerative or post-traumatic conditions [188].

Research conducted by Wang and colleagues [189] demonstrated that viral vector-mediated gene transfer altered the cartilage-forming, and fat-forming capabilities of IPFSCs, affecting mitotically exhausted IPFSCs proliferation rates and lineage commitment preferences. These observations suggest that both acellular matrix cultivation and life-extension methodologies may be employed for proliferation-exhausted adult stem cells’ proliferation and cell-type specialization, representing an opportunity for future cellular-mediated tissue repair therapies [189].

Preconditioning represents a promising strategy for enhancing the therapeutic potential of extracellular vesicles derived from IFPSCs in KOA management [190,191].

Specifically, research conducted by Wu and colleagues [192] demonstrated that TNF-a priming enhanced extracellular vesicle production from IFPSCs compared to non-primed cells (via activation of the Phosphoinositide 3-kinase/Protein Kinase B, PI3K/AKT, signaling cascade), improving therapeutic efficacy in relieving disease-associated joint alterations in murine OA models.

## 4. Discussion

This review presents an in-depth evaluation of novel therapeutic approaches targeting the IFP in KOA, with particular focus on their impact on inflammatory mediators, fibrotic remodeling processes, and nociceptive sensitization. By integrating findings from both preclinical models and translational research, we highlight the pivotal role of the IFP in the pathophysiological mechanisms underlying OA and examine translational relevance for patient care.

It has been studied how, in patients with OA, the IFP not merely represents a fat storage and a biomechanical shock absorber, but also an active inflammatory organ, displaying hypertrophic adipocytes, infiltration of pro-inflammatory M1 macrophages, and a senescence-associated secretory phenotype (SASP) [38,54,55].

This structure also stimulates the secretion of prostaglandins such as PGE2, PGF2a, and PGD2, which contribute, through paracrine stimulation, to the development of a chronic inflammatory trigger in the synovium and cartilage of adjacent tissues [193].

Among the pathological changes in the IFP in KOA, fibrotic remodeling is the most important, predominantly driven by the TGF-β signaling pathway and profibrotic M1 macrophages [194]. These structural changes alter the biomechanical properties of the tissue, promoting a change in loading distribution and thus supporting degeneration of joint structures [64].

The IFP is also a densely innervated structure, influencing pain stimulation [69]. In KOA, increased expression of CGRP and substance P within IFP gives rise to a neurogenic inflammatory loop that contributes to the transition from acute to chronic OA pain [69,70].

Several treatments have been developed specifically to target the IFP, ranging from pharmacological therapies to interventional procedures. The use of nonsteroidal anti-inflammatory drugs, particularly celecoxib, has demonstrated efficacy in inhibiting prostanoid production, particularly in patients with significant inflammatory profiles [80]. However, inflammatory patterns are highly heterogeneous, so phenotyping of the IFP may be useful to establish personalized therapy and achieve improved therapeutic outcomes [195,196].

LIPUS, a minimally invasive technique, shows therapeutic potential in reducing IFP fibrosis through several mechanisms, such as HIF-1 inhibition and macrophage phenotypic modulation [110,197]. Specifically, this minimally invasive approach inhibits TGF-β signaling and stimulates macrophage differentiation into M2, reducing the population of pro-inflammatory M1 macrophages [112].

Gene therapy has also shown promising results, demonstrating how CCL2 receptor antagonists can suppress OA-related histopathological changes in the IFP, reducing macrophage infiltration into synovial tissues and suppressing additional intrinsic chemokine production [82]. Intra-articular injective therapies have also demonstrated efficacy, particularly when used in combination (e.g., PRP with HA), thanks to a synergistic mechanism that simultaneously modulates inflammation and biomechanical function [198]. However, the limited duration of the therapeutic effect, approximately 9 months, should prompt clinicians to consider treatment cycles rather than single administrations [198].

Genicular artery embolization has been associated with functional improvement in 70–80% of patients, suggesting a potential role for treating the vascular component that contributes to chronic pain through the ablation of neovascularized areas [163].

Regarding the surgical approach, studies indicate that selective excision of the IFP may help reduce the progression of KOA; however, there is no conclusive evidence supporting the removal of this structure during knee replacement surgery in patients with KOA, and high-quality studies highlighting the best surgical strategy are lacking [105,106].

The most innovative therapeutic approach involves the use of IFP-derived mesenchymal stromal cells, which have a natural tissue regenerative ability. These cells exhibit enhanced chondrogenic potential than other mesenchymal populations, suppress apoptosis, and stimulate matrix production, contributing to reduce the rate of OA progression [182,199,200].

Despite these advances, significant gaps persist in our knowledge and capacity to therapeutically target the IFP.

The pathological involvement of the IFP varies significantly among patients [10], and it would be important to identify biomarkers to stratify patients, working toward patient-specific treatment. Novel imaging tool able to detect more accurately the level of tissue inflammation, not just structural characteristics, are also needed. Moreover, the time course of IFP pathology during disease development has not been fully defined, limiting optimization of intervention timing [65].

Additionally, the predominance of rodent models in translation of preclinical findings may not fully capture the complexity of human IFP pathology, particularly regarding chronic OA development and the impact of comorbidities.

Future research must give priority to the development of non-invasive IFP phenotyping methods, advanced imaging techniques, and artificial intelligence-enhanced analysis to promote a personalized therapeutic approach.

## 5. Conclusions

In conclusion, the IFP emerges as a promising therapeutic target in OA management, offering multiple intervention opportunities across the spectrum of conservative, minimally invasive, and surgical approaches. The convergence of mechanistic understanding, preclinical efficacy data, and early clinical observations supports the continued investigation of IFP-targeted therapies. However, the translation of these insights into standardized clinical practice requires more standardized studies like randomized controlled trials specifically designed to evaluate IFP-targeted interventions, the development of reliable biomarkers for patient selection and the creation of algorithms that consider IFP assessment in routine OA management protocols.

## Figures and Tables

**Table 1:** Conservative Therapies. Summary of conservative therapeutic strategies for KOA, focusing on early and preventive interventions targeting inflammation, fibrosis, and biomechanical dysfunction. Treatments include pharmacological agents, lifestyle modifications and gene-based approaches, all aiming to modulate key pathological mechanisms in the IFP and surrounding joint environment.

Therapeutic Strategy	Mechanism of Action	OA Stage	Therapeutic Target
Anti-inflammatory drugs (e.g., Celecoxib) [80]	Suppression of prostanoid production in IFP	Early to moderate	Inflammatory mediators in IFP
LIPUS [81]	Modulates macrophage phenotypes, reduces TGF-β expression, fibrosis, and inflammation	Early to moderate	Fibrosis and macrophage-mediated inflammation
Gene Therapy (7ND) [82]	Blocks CCL2/CCR2 axis to prevent monocyte/macrophage infiltration and fibrosis	Early	Inflammation and fibrosis pathways in IFP
Diet modification [83,84]	Reduces expression of NF-κB, IL-1, IL-6, TNF-α, and COX-2; mitigates inflammation	Preventive	Systemic and local inflammatory signaling
Physical therapy [85,86]	Maintains IFP mobility and volume, prevents atrophy and fibrosis	Early	Biomechanical protection and fat pad integrity

**Table 2:** Minimally Invasive Treatments. Overview of minimally invasive treatment strategies for KOA, highlighting therapeutic mechanisms, target tissues, and applicable disease stages. Approaches such as intra-articular injections, neuro-modulatory techniques, and vascular interventions address key pathological processes including inflammation, fibrosis, neo-innervation, and nociceptive signaling.

Therapeutic Strategy	Mechanism of Action	OA Stage	Therapeutic Target
Hyaluronic Acid (HA) injection [87,88]	Viscosupplementation, chondrocyte support, anti-inflammatory	Early to moderate	Synovial fluid and cartilage
Platelet-rich Plasma (PRP) [89,90]	Releases growth factors, reduces CGRP-positive nerve fibers	Early to moderate	Nociceptive fibers, cartilage, IFP inflammation
Corticosteroids [91,92,93]	Modulate immune cells, inhibit TGF-β and matrix enzymes	Early	IFP inflammation, fibrosis, nerve sensitization
CNP injection [94]	Inhibits TGF-β signaling, reduces fibrosis and pain	Early to moderate	Fibrosis and chronic pain in IFP
Electroacupuncture (EA) [95,96,97,98]	Suppresses NLRP3 inflammasome, reduces fibrosis and inflammation	All stages	IFP fibrosis, synovitis
Genicular Artery Embolization (GAE) [99,100]	Occludes abnormal neovessels to reduce inflammation and innervation	Moderate to severe	Neovascularization and pain circuits
Genicular Nerve Radiofrequency Ablation [101,102]	Disrupts nociceptive nerve conduction	Moderate to severe	Articular pain pathways

**Table 3:** Surgical therapy. Surgical resection of the IFP as a therapeutic strategy for moderate to severe KOA. This intervention targets fibrotic and inflamed IFP tissue, aiming to reduce inflammation and alleviate pain by modifying IFP structure and its pathological contribution to OA progression [103,104].

Therapeutic Strategy	Mechanism of Action	OA Stage	Therapeutic Target
Surgical resection of IFP [105,106]	Removes fibrotic/inflamed IFP tissue, reduces inflammation	Moderate to severe	IFP structure and inflammation

## Data Availability

No new data were created or analyzed in this study.

## Abbreviations

The following abbreviations are used in this manuscript:

ACL	Anterior Cruciate Ligament
AFU	Anatomo-Functional Unit
BMI	Body Mass Index
BMP-7	Bone Morphogenetic Protein-7
BMP-14	Bone Morphogenetic Protein-14
CCL2	Monocyte chemoattractant protein-1
CCLD	Cranial Cruciatus Ligament Disease
CCR2	C-C Motif Chemokine Receptor 2
CCS	Corticosteroids
CNP	C-type natriuretic peptide
DAMPS	Damage-Associated Molecular Patterns
EA	Electroacupuncture
FABP4	Fatty Acid-Binding Protein-4
GAE	Genicular Artery Embolization
GFs	Growth Factors
HA	Hyaluronic Acid
HIF-1	Activation of hypoxia-inducible factor 1
IFP	Infrapatellar Fat Pad
IFPSCs	Infrapatellar Fat Pad-derived Stem Cells
IL-6	Interleukin-6
IL-1	Interleukin-1
iNOS	Inducible Nitric Oxide Synthase
KOA	Knee Osteoarthritis
LIPUS	Low-intensity pulsed ultrasound
MCP-1	Monocyte chemoattractant protein-1
miR100-5p	MicroRNA 100-5-p
MSCIPFP-Exos	Mesenchymal-derived extracellular vesicles from the infrapatellar adipose tissue
MMP-1	Matrix Metalloproteinase 1
MMP-3	Matrix Metalloproteinase 3
MPA	Methylprednisolone acetate
MRI	Magnetic Resonance
m-TOR	Mechanistic Target of Rapamycin
NF-κB	Nuclear factor kappa light chain enhancer of activated B cells
NLRP3	NLR family pyrin domain containing 3
NO	Nitric Oxide
NRS	Numeric Rating Scale
OA	Osteoarthritis
PGE2	Prostaglandin E2
PGF2α	Prostaglandin F2α
PGD2	Prostaglandin D2
PI3K/AKT	Phosphoinositide 3-kinase/Protein Kinase B.
PPAR-γ2	Peroxisome Proliferator-Activated Receptor Gamma 2
PPTs	Pressure Pain Thresholds
PRP	Platelet-Rich Plasma
RFA	Radiofrequency Ablation
RUNX2	Runt-related transcription factor 2
SASP	Secretory phenotype
SM	Synovial Membrane
SMAD3	Mothers against decapentaplegic homolog 3
SOX-9	SRY-related HMG-box gene 9
TE	Tropoelastin
TGF-β	Transforming growth factor-β
TIMP	Tissue Inhibitor of Metalloproteinases
TNF-α	Tumor necrosis factor
VAS	Visual Analog Scale
VCAM1	Vascular Cell Adhesion Molecule 1
VEGF	Vascular endothelial growth factor
WOMAC	Western Ontario and McMaster Universities Osteoarthritis Index
